# Recent trends and future directions for lung cancer mortality in Europe

**DOI:** 10.1038/sj.bjc.6600352

**Published:** 2002-07-15

**Authors:** P Brennan, I Bray

**Affiliations:** Unit of Environmental Cancer Epidemiology, International Agency for Research on Cancer, 150 cours Albert-Thomas, 69372 Lyon cedex 08, France; Department of Mathematics and Statistics, University of Plymouth, Drake Circus, Plymouth, PL4 8AA, UK

**Keywords:** lung cancer, trends

## Abstract

Lung cancer mortality patterns throughout Europe are very heterogeneous and largely reflect past smoking habits. In order to clarify the changing patterns of lung cancer in Europe we have plotted the overall lung cancer trends among men and women for 20 countries from 1950 up to1998. Furthermore, using a Bayesian age-period-cohort approach, we have calculated 5 year projections of lung cancer rate up to 2003. Finally, we make some comments on probable future trends by analysing recent trends in adults aged <55 years. Lung cancer mortality rates up to age 75 years portray a general trend of decreasing lung cancer rates among men and increasing lung cancer rates among women. Exceptions to this decrease among men include Hungary where not only are current mortality rates much higher than previously observed in any other country (at 76.7 out of 100 000 in 1998) but they are projected to increase further in the short term. Rates among adults aged <55 years have recently peaked, indicating that overall rates are likely to peak in the next decade. Among women, rapid increases have been observed in Denmark, Netherlands, Hungary, Ireland and UK. Whereas Ireland and UK rates have started to decrease and are projected to continue falling, rates in the other three countries are projected to increase further. Trends in women aged <55 years indicate that rates in Danish women will peak in the next decade, whereas lung cancer rates among Dutch women are likely to continue increasing. Rates in Hungarian women are likely to increase and will surpass the current high rate observed in Denmark.

*British Journal of Cancer* (2002) **87**, 43–48. doi:10.1038/sj.bjc.6600352 www.bjcancer.com

© 2002 Cancer Research UK

## 

Lung cancer is the most common cause of cancer mortality among men in Europe, and is becoming an increasingly important cause of cancer mortality among women (Bray *et al*, 2002). Lung cancer mortality patterns throughout Europe are very heterogeneous and largely reflect past smoking habits (Kubik *et al*, 1995), although changes in cigarette composition are also likely to be important. In order to clarify the changing patterns of lung cancer in Europe we have plotted the overall lung cancer trends among men and women for 20 countries from 1950 up to the most recent collection of data, usually 1996–1998, depending on each country. Furthermore, using a recently validated Bayesian age-period-cohort approach, we have calculated 5 year projections of lung cancer rate up to 2001–2003. Given the delay of several years in obtaining cancer mortality data these short term projections serve as a measure of current lung cancer mortality. Finally, as changes in lung cancer rates tend to be observed first among younger age groups, we make some comments on likely future trends by analysing recent trends in adults aged less than 55 years.

## METHODS

Official lung cancer mortality figures for 20 European countries, stratified for sex and 5 year age groups, were obtained from the WHO mortality database for the years 1950–1998. Information from other European countries over this time period was not available. Information from the former-Czechoslovakia was not available after 1991, and lung cancer mortality for 1992–1998 was estimated using data from the Czech Republic. Due to the unreliability of death certification in the very old, age groups from 75 years and above were excluded. Age and sex specific estimates of the population in each country were also obtained.

Short-term mortality projections were made using a generalised Bayesian age-period-cohort model (Bashir and Esteve, 2001; Bray, 2002; Knorr-Held and Rainer, 2001). Age-period-cohort models simultaneously estimate the increasing risk of mortality with age, cohort effects (factors affecting a group of people born around the same time) and period effects (which apply to all people at a certain point in time). Cohort effects arise when different birth cohorts have different levels of exposure to a particular risk factor (e.g. cigarette smoking). Temporal changes which affect all members of the population (e.g. air pollution, a change in cigarette composition or a new treatment) are examples of period effects which could affect cancer mortality rates. Identifying individual cohort, period or age effects is impossible with making further assumptions, although this identifiability problem is not of concern when making projections.

Projections based on extrapolating classical estimates of age, period and cohort effects require the analyst to make parametric assumptions (Osmond, 1985) and are sensitive to variation in the most recent cohort effects, which can be unstable due to small numbers. The Bayesian approach presented here uses non-parametric smoothing to reduce variation, hence improving the precision of the projections, and allows us to incorporate *a priori* belief about the smoothness of the age, period and cohort effects (Berzuini and Clayton, 1994). This is achieved by specifying an autoregressive prior model which smoothes effects on each time scale, preventing parameter estimates from differing too much from those in adjacent time bands. The expected value for each effect is based on an extrapolation from its two immediate predecessors. The autoregressive model extrapolates period and cohort effects forward to make projections for rates in future periods, (age effects are not extrapolated since the age groups of interest in future periods do not change). Problems in identifying and interpreting individual parameters, caused by the linear relationship between age, period and cohort effects in these models, do not affect projected rates.

Five year age-standardised fitted and projected rates were estimated from samples of 15 000 drawn from the posterior distribution, taking the median as an overall summary rate. 90% credible intervals were calculated as the 5th and 95th percentiles of the samples (a credible interval may be interpreted as a confidence interval calculated using Bayesian techniques). All age-standardised mortality rates were calculated using the world standard truncated at age 75 years (Parkin *et al*, 1997). Further information on methods and implementation are described elsewhere (Bashir and Esteve, 2001; Bray, 2002; Knorr-Held and Rainer, 2001).

This method has been compared with three alternative models for both incidence and mortality data and a variety of different cancer sites by comparing the performance of empirical projections (Bashir and Esteve, 2001; Bray, 2002). Overall, the Bayesian projections outperformed all other methods considered. This method is also robust and was found to work well in situations where other approaches failed, such as when the numbers of observed cases are very small.

## RESULTS

**Figure 1:**
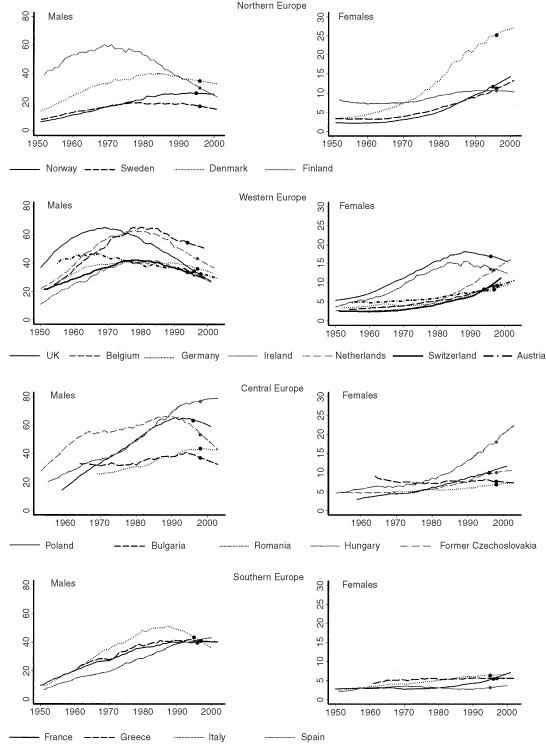
Age standardised rates (0–74 years). The y axis represents standardised rates per 100 000 and the *x* axis represents calendar year. Points mark the end of the observed data and the beginning of the 5-year projections.

Lung cancer mortality rates up to age 75 years portray a general trend of decreasing lung cancer rates among men and increasing lung cancer rates among women (Figure 1

and Table 1

**Table 1:**
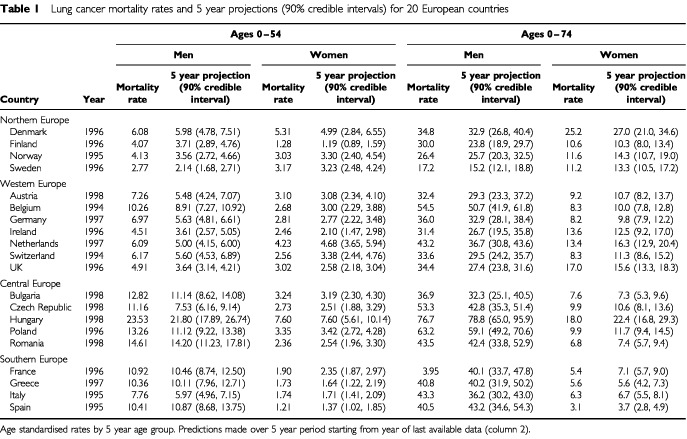
Lung cancer mortality rates and 5 year projections (90% credible intervals) for 20 European countries

). Among men, countries in which lung cancer rates peaked early, including Finland, UK, Belgium and The Netherlands, reported peak mortality rates of approximately 60 out of 100 000, although these have since substantially declined, and short term projections confirm that these declines are likely to continue. Other countries in Northern and Western Europe have also peaked, although at much lower levels, and some decline in incidence is now evident. In southern Europe, rates among men have peaked in Italy at a moderate level and are now decreasing, whereas rates are constant in Greece and France, and gradually increasing in Spain. In Central Europe a very heterogeneous picture is observed, with Romania and Bulgaria having moderate rates similar to Southern Europe. Rates in Poland and Czech Republic both peaked during the 1990s at very high levels, although a decrease is now evident in both countries, especially in Czech Republic. Mortality rates in Hungary are however surprising. Not only are current mortality rates (76.7 out of 100 000, age-adjusted for age group 0–74, in 1998) much higher than previously observed in any other country but they are projected to increase further in the short term. The current male lung cancer mortality rate in Hungary represents the highest national lung cancer mortality rate ever observed.

Lung cancer mortality rates among women over the last 50 years have increased in most countries (Figure 1 and Table 1). In Northern Europe, a very rapid increase in lung cancer mortality rates has been observed in Denmark, which now has the highest female national mortality rates in the world. Short term projections do not indicate that this mortality rate is about to peak. In Western Europe, high mortality rates observed in UK and Ireland have peaked and are now decreasing. In contrast, female lung cancer mortality in The Netherlands is rapidly increasing and is projected to surpass UK and Ireland rates. In Central Europe, a dramatic increase has been observed in Hungary and is projected to further increase in the short term. Elsewhere in Central and Southern Europe, lung cancer rates are still relatively low, although the beginning of the lung cancer epidemic may be observed in several countries including Poland, Czech Republic and France.

**Figure 2:**
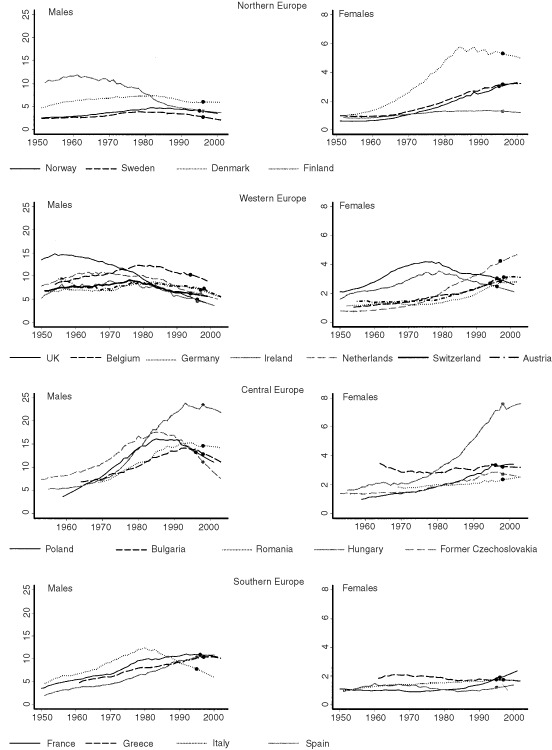
Age standardised rates (0–54 years). The y axis represents standardised rates per 100 000 and the *x* axis represents calendar year. Points mark the end of the observed data and the beginning of the 5-year projections.

Age standardised rates up to age 54 years indicate that usually, a sharp decline in the middle age may anticipate by about 10–15 years a decline in mortality rates overall. For example, male and female rates up to age 55 years in the UK peaked around 1955 and 1975 respectively (Figure 2

), whereas the rates up to age 75 years peaked around 1970 and 1990 respectively (Figure 1). Similar trends may be seen in Finnish, Dutch, Polish and Italian men, where overall decreases are evident, as well as among Irish women. Such a relationship is expected, as lung cancer rates are strongly related to previous cohort changes in smoking practices and will first become apparent when the cohort is in middle age. Even so, this relationship is not evident in all cases and, for example, among Belgian and Czech men, declines in middle age and overall tended to occur almost concurrently, indicating that period effects related to smoking or other factors also play a role.

Those countries which have seen a sharply increasing lung cancer mortality rate which are projected to increase at least in the short term and which are therefore of major concern include Hungarian men, and Danish, Dutch and Hungarian women. Rates in Hungarian men aged up to 55 years are likely to have peaked and a slight decline is projected in the short term, indicating that overall rates are likely to peak soon, although at a very high level (Figure 2 and Table 1). Similarly, among Danish women rates have peaked in the age group up to 55 years and are currently decreasing, indicating that overall lung cancer rates should soon peak close to their current high level, probably at around 30 out of 100 000, and start to decrease. In Hungary, female lung cancer mortality rates have shown a constant increase up to 1998, although 5 year projections indicate that they may have peaked at their current high level (7.6 out of 100 000). The 90% credible interval of (5.61, 10.14) is wide and it is not possible to make firm predictions regarding Hungarian female lung cancer rates in the medium term. However, rates among Hungarian women aged less than 55 years are substantially higher than the maximum rate observed among Danish women in this age group, indicating the mortality rate among all Hungarian women is likely to exceed the current high rates observed among Danish women. Finally, in The Netherlands a constant increase has been observed in younger women indicating that the overall increase is likely to continue.

## DISCUSSION

In summary, age standardised lung cancer mortality rates among men have peaked or are decreasing in all European countries included in this analysis except for Hungary, and possibly Spain. Among adults aged less than 55 years, mortality rates in both Spain and Hungary appear to be in the process of peaking, indicating that a peak in the overall rate is likely to occur in the next 10 years. While the peak mortality rate in Spain will occur at a relatively modest level, the peak in Hungarian rates, when it occurs, is are likely to represent the highest national lung cancer mortality rate observed for any country. Among women, although rates are decreasing in UK and Ireland, it is likely that substantial increases will be observed for most other countries.

Trends in lung cancer mortality rates are known to occur largely as a result of cohort effects in specific populations, which is almost entirely related to practices in cigarette smoking. In theory, it may be possible to improve the predictive accuracy of these models by incorporating past information on cigarette consumption. Information is available for the proportion of adult smokers among men and women in each country from the WHO ‘Health For All’ database and is included in Table 2

**Table 2:**
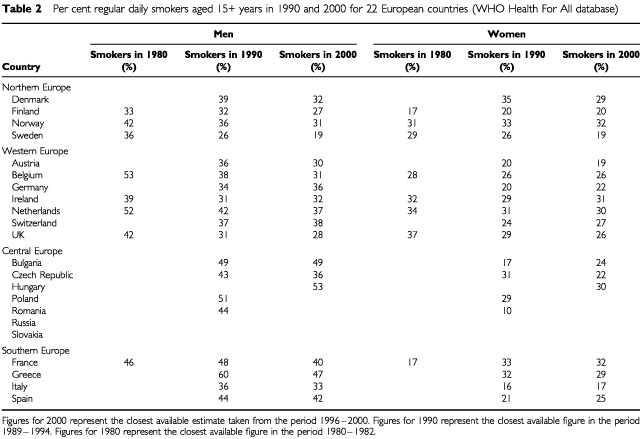
Per cent regular daily smokers aged 15+ years in 1990 and 2000 for 22 European countries (WHO Health For All database)

for all countries in the current analysis along with Slovakia and Russia. However, accurate age specific information on smoking prevalence would be required if this information were to be incorporated into the prediction model and this information is not available. The smoking prevalence data do however indicate that overall smoking has decreased among men in nearly all countries in Northern, Western and Southern Europe. In Central and Eastern Europe, a substantial decrease has been observed in Czech Republic which is balanced by an identical increase in neighboring Slovakia. Whether this is due to methodological differences in the surveys or represents a real phenomenon is not clear. A large increase in smoking prevalence has also been observed among Russian men, whereas survey information at two different time points was not available for Hungary, Poland and Romania. Among women a decrease in tobacco consumption has been observed in several countries including Denmark, Sweden, Czech Republic and UK, whereas constant or increasing tobacco consumption patterns have been observed for other countries.

These trends illustrate the large potential for tobacco control on lung cancer mortality. The evidence from the two countries where the lung cancer epidemic first peaked (UK and Finland) is that if anti-smoking policies are adopted which result in cessation of smoking among a large proportion of current smokers then these trends can be reversed. Five-year projections of lung cancer mortality among men in UK and Finland indicate that current rates in these two countries are likely to be among the lowest in Europe, with only Norway and Ireland having comparable rates and Sweden having substantially lower rates. However, such a decline in lung cancer rates is not inevitable and is unlikely to occur in the absence of strong public health campaigns, which encourage current smokers to quit smoking. Although there are potential gains to be made in all countries in terms of reducing tobacco consumption, a clear priority for such work is in Central Europe, which has recently seen massive investment by foreign tobacco companies.
